# Who chooses telehealth? A cross-sectional cohort study of diabetic patients in outpatient clinic follow-up

**DOI:** 10.1371/journal.pdig.0001545

**Published:** 2026-07-09

**Authors:** Arkers Kwan Ching Wong, Luna Ziqi Liu, Frances Kam Yuet Wong, Jun Liang, Danny Wah Kun Tong, Man Li Chan, Man Kin Wong, Bo Chu Wong, Yeuk Sze Tang, Wai Hing Ho, Sau Ching Chiang

**Affiliations:** 1 School of Nursing, The Hong Kong Polytechnic University, Hung Hom, Hong Kong; 2 The Joint Research Centre for Primary Health Care, The Hong Kong Polytechnic University, Hung Hom, Hong Kong; 3 New Territories West Cluster, Hospital Authority, Tuen Mun, Hong Kong; 4 Head Office, Hospital Authority, Homantin, Hong Kong; Hadassah Academic College, ISRAEL

## Abstract

Telehealth, the remote delivery of healthcare services via digital communication technologies, effectively manages diabetes, yet factors influencing its use—particularly in primary care—remain underexplored. This cross-sectional study investigates factors affecting participation and continuity of telehealth services among 1,094 diabetic patients in General Outpatient Clinics (GOPC). Two dependent variables—participation and continuity—were assessed at recruitment (T1) and post-program (T3). Socio-demographic, clinical, and eHealth-related characteristics served as independent variables. Descriptive statistics and binary logistic regression were utilized, including interaction terms for moderation effects. Key findings indicated that initial participation was significantly associated with eHealth literacy, health-related internet use, and mobile technology confidence. For continuity, significant predictors included age, care frequency, and income source, with care frequency’s positive effect diminishing as eHealth literacy increased. Overall, this study highlights the importance of digital capabilities and household support. Guided by the Unified Theory of Acceptance and Use of Technology 2 (UTAUT2), the results suggest that certain UTAUT2 constructs, such as effort expectancy and facilitating conditions, may be particularly relevant for understanding telehealth engagement in this context.

**Trials Registration:**

The study was registered on ClinicalTrials.gov (Identifier: NCT05183685) on 10 Dec 2022.

## Author summary

Telehealth can make diabetes follow-up more convenient, but it only works well if patients are willing and able to use it. In this study, we examined who chose to join a video-based telehealth follow-up program and who continued using it over time among patients with diabetes attending public general outpatient clinics in Hong Kong. We found that patients with stronger digital skills, more confidence using mobile devices, and more experience seeking health information online were more likely to join. Continued use was lower among older patients and among those relying on family support or pensions as their income source, while patients with more frequent help at home were more likely to continue. We also found that patients with stronger digital skills appeared to rely less on household help to stay engaged. These findings suggest that telehealth programs should not focus only on making services available. They also need simple instructions, practical onboarding, and support for patients who are older, less digitally confident, or have limited help at home.

## Introduction

Diabetes mellitus (DM) is a widespread chronic condition that necessitates ongoing monitoring and management, with over 95% of the 537 million people worldwide living with DM requiring regular follow-up care [1,2]. In Hong Kong, approximately 500,000 patients have been receiving diabetes care at Government-funded General Outpatient Clinics (GOPC) since 2019 [3], contributing to increasing service pressure, overcrowding, and prolonged waiting times. As patient numbers continue to rise, the reliance on in-person follow-up visits has become increasingly challenging for both healthcare providers and patients [4]. For individuals with chronic conditions—particularly older adults and those with mobility limitations—frequent clinic attendance may also impose considerable logistical and physical burdens [5,6]. These challenges highlight the need for alternative follow-up models that can maintain continuity of care while reducing dependence on face-to-face outpatient services.

Telehealth, defined in this study as synchronous video-based consultations between patients and nurses via secure platforms [7], has emerged as a promising solution. By providing a convenient and flexible alternative to traditional outpatient follow-up, this model effectively reduces the strain on in-person services [8]. Existing evidence indicates that telehealth-based follow-up is clinically feasible and effective for diabetes management, with outcomes such as glycated haemoglobin (HbA1c) control comparable to those achieved through traditional face-to-face consultations [9]. HbA1c reflects average glycaemic control over the preceding three months and is widely accepted as the gold standard for monitoring long-term diabetes outcomes. For patients who must balance healthcare needs with work commitments, the online format of telehealth consultations offers flexibility that accommodates their schedules [10]. Furthermore, telehealth is particularly well-suited for follow-up consultations involving stable and early-stage cases, which are primarily focused on reviewing blood glucose levels and not requiring real-time physical assessments [11]. These findings underscore telehealth’s adaptability within the primary care setting and its potential to enhance patient convenience while improving overall healthcare system efficiency.

Despite evidence indicating that telehealth maintains clinical effectiveness comparable to traditional in-person visits for diabetes management, as well as its numerous benefits in alleviating the burden on the healthcare system and enhancing patient convenience, its adoption remains low. Even in the United States, which has one of the highest global rates of telehealth adoption for diabetes management, only 38.7% of diabetic patients choose to use this service for their follow-up needs, suggesting that significant barriers and gaps still exist in the practical widespread adoption and utilization of telehealth in diabetes care [12]. While barriers within the healthcare delivery process have garnered significant attention and research, contributing to continual optimization of service provision, the factors influencing patients’ personal preferences and decisions—who are the recipients and decision-makers of these services—remain underexplored [13]. Understanding diabetic patients’ preferences for telehealth follow-up care provides valuable insights into their concerns when selecting a follow-up model, which reveals both the strengths and limitations of current telehealth services in addressing diverse patient needs. Notably, age and gender have emerged as consistent predictors of telehealth usage, particularly during the post-pandemic shift toward telehealth. Older adults may face greater difficulties with technology use, while women have been found to use telehealth tools more actively, possibly due to health-seeking behaviour patterns [14,15]. Investigating these demographic factors helps elucidate the nuanced barriers different subgroups may face.

In particular, patients’ digital engagement and capabilities may strongly influence their willingness to use telehealth. For example, eHealth literacy—defined as the ability to seek, understand, and apply electronic health information—is essential for navigating telehealth platforms [16,17]. Similarly, mobile technology confidence and prior use of the Internet for health purposes (health-related internet use) reflect users’ readiness to interact with telehealth. Beyond individual digital factors, structural support such as the availability of household assistance (measured by care frequency) may also influence continuity of telehealth use, especially among older adults or those with limited autonomy [18]. However, despite growing international evidence on telehealth adoption, there remains limited empirical understanding of how digital capabilities, household support, and socio-demographic factors jointly influence both initial participation and continuity of telehealth use within Hong Kong’s primary care setting.

To address this knowledge gap, the present study collected and analysed demographic, care frequency, and digital usage data from patients with diabetes attending GOPCs in Hong Kong to identify factors associated with both the initial adoption and continued use of telehealth.

## Methods

### Ethics statement

This study received IRB approval from both the Hospital Authority (HA) (REC Ref. No.: 20119, The Joint NTWC REC) and the Hong Kong Polytechnic University (No. HSEARS20200619003). Additionally, it was registered on ClinicalTrials.gov (Identifier: NCT05183685) on 10 Dec 2022.

### Study setting and data collection

#### Study setting.

This cross-sectional study utilized data collected from the Telehealth Consultation at GOPC Program specifically designed for diabetic patients. The telehealth program was implemented across seven outpatient clinics within the New Territories West Cluster in Hong Kong, collectively serving over 50,000 diabetic patients annually [19]. A full description of the program is available elsewhere [20]. The Strengthening the Reporting of Observational studies in Epidemiology (STROBE) research checklist (S1 STROBE Checklist) was utilized to report the findings of this study [21]. Given the cross-sectional nature of the analysis, causal relationships between predictors and telehealth participation or continuity cannot be inferred.

The Telehealth Consultation Program consisted of scheduled, nurse-led follow-up consultations conducted via a secure video-conferencing platform (e.g., Zoom), designed to complement routine diabetes care in General Outpatient Clinics. All telehealth consultations were conducted using **real-time video communication**, and no phone-only or asynchronous messaging consultations were included in the program to ensure that all participants received the benefits of visual interaction, such as nonverbal cues, on-screen demonstrations, and enhanced patient-provider engagement. Eligible patients received structured telehealth sessions focusing on diabetes self-management support, including review of blood glucose records, medication adherence, lifestyle modification, and individualized health education. Consultations were delivered by trained physicians and advanced practice nurses, with each session typically lasting 20–30 minutes. The program emphasized patient engagement, continuity of care, and flexibility, allowing consultations to be conducted remotely while maintaining clinical oversight and documentation within standard outpatient workflows.

### Participants and data collection

Participants for the current study were recruited from a larger Telehealth Consultation Program implemented in GOPCs for patients with diabetes. While the telehealth consultation program was designed as a service delivery intervention, the present study represents a secondary analysis of data collected from patients who were screened and assessed for eligibility at baseline. Research assistants introduced the program, explained its objectives, benefits, and potential risks, and conducted baseline data collection. The inclusion criteria for participants were: (1) aged 18 or older, (2) diagnosed with diabetes, (3) attending regular follow-up appointments, and (4) capable of using a smartphone or computer, or having assistance available. Participants were required to be capable of providing informed consent. Exclusion criteria included: (1) patients with dementia, (2) patients with unaccompanied hearing or vision loss, (3) lack of internet access.

A total of 1,094 patients met the eligibility criteria and provided baseline data. Socio-demographic, clinical, and eHealth-related data were collected prior to program invitation. These eligible participants were then introduced to the telehealth program, including a detailed explanation of its objectives, benefits, and potential risks.

Of the eligible participants, 204 declined to participate, while 890 agreed and were enrolled. Enrolled participants were then randomized into two arms: the intervention group (n = 410) received nurse-led telehealth consultations, while the control group (n = 480) continued with standard in-person follow-up care at General Outpatient Clinics, consistent with usual practice. Although participants were randomized to intervention and control groups in the parent trial, this study focused solely on analysing factors influencing participation and continuity within the telehealth group; the control group was not included in the current analyses.

**Fig 1:**
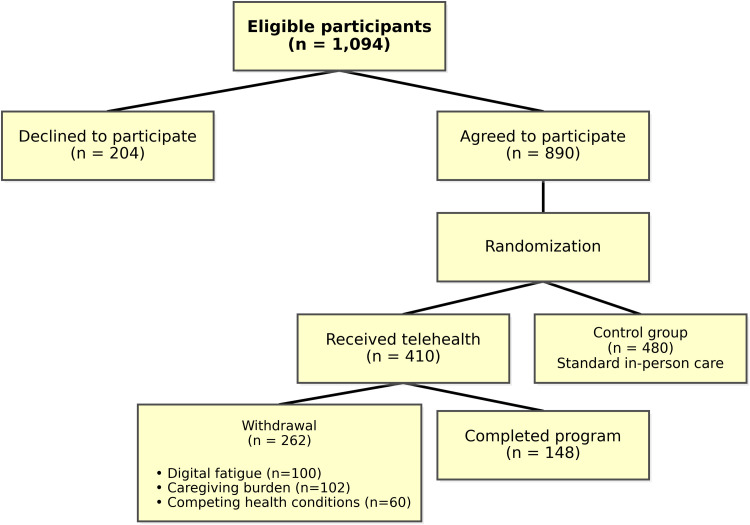
Process of samples’ allocation and grouping.

Participants enrolled in the telehealth group were followed longitudinally at 42 weeks (T2) and 84 weeks (T3). Withdrawal from the telehealth program was documented by research staff at the time of discontinuation. Reasons for withdrawal were recorded descriptively based on participant self-report or clinical documentation and included lack of interest, competing caregiving responsibilities, and personal matters. Withdrawal reasons were used for descriptive reporting only and were not included as predictors in the regression analyses. The allocation and grouping process is presented in Fig 1.

To ensure participant privacy, all direct personal identifiers were removed from the dataset prior to analysis. To strictly maintain anonymity, especially within small subgroups, any stratifications yielding fewer than five individuals were aggregated or suppressed in the final reporting. While the raw dataset cannot be made public due to privacy restrictions, the statistical analysis code (SPSS syntax) used to generate the findings is available from the corresponding author upon request.

### Theoretical framework

In this study, the Unified Theory of Acceptance and Use of Technology 2 (UTAUT2) was employed as a conceptual framework to guide variable selection and interpretation, rather than as a formal measurement instrument. UTAUT2 was selected as a guiding framework because it specifically focuses on technology adoption and continued use, which aligns with the study’s emphasis on telehealth engagement rather than clinical self-management behaviour. Unlike disease-specific behavioural models, UTAUT2 emphasizes digital capability, facilitating conditions, and usage persistence. UTAUT2 encompasses seven constructs related to users’ behavioural intentions and technology use including Performance Expectancy (perceived usefulness), Effort Expectancy (ease of use), Social Influence, Facilitating Conditions, Hedonic Motivation, Price Value, and Habit. These constructs have been widely applied in healthcare and telemedicine research, supporting its relevance as an interpretive framework for understanding telehealth engagement [22–24].

Guided by UTAUT2, we examined how socio-demographic characteristics, eHealth-related attributes (including mobile technology confidence, eHealth literacy, and health-related internet use), and care frequency influence telehealth engagement. Specifically, these variables were conceptually aligned with relevant UTAUT2 constructs to support theory-informed interpretation. Mobile technology confidence and health-related Internet use were considered in relation to Performance Expectancy and Effort Expectancy, while care frequency was interpreted in relation to Social Influence. eHealth literacy was associated with Facilitating Conditions, and occupation status and income source were linked to Price Value [24,25]. Habit was not directly measured but partially reflected in continuity behavior [25].

**Fig 2:**
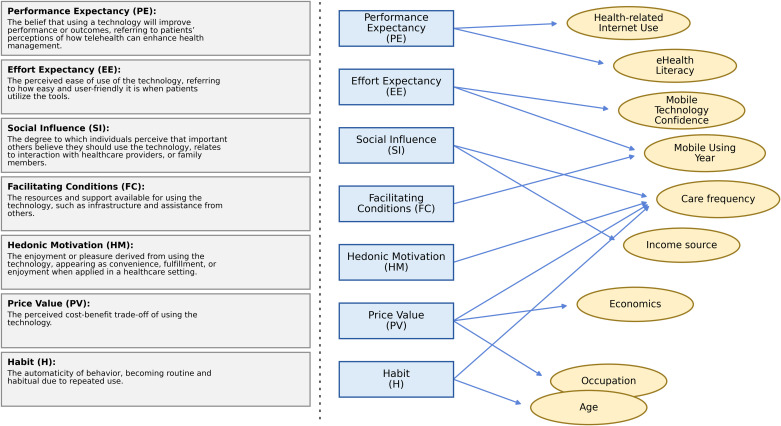
Selection and mapping of UTAUT2 constructs to study variables, illustrates the conceptual alignment between UTAUT2 constructs and study variables, which informed our model specification and variable selection in the binary logistic regression analysis.

Due to the pragmatic, service-based design of the telehealth program and reliance on routinely collected data, the validated UTAUT2 questionnaire was not administered. Accordingly, the UTAUT2 was applied as a conceptual framework rather than being operationalized a formal model for construct testing or validation. This study does not aim to operationalize, validate, or test UTAUT2 constructs (Fig 2).

### Research questions and hypotheses

This study addressed two research questions:

Question 1 (Q1)—What factors influence diabetic patients’ willingness to participate in telehealth programs?

Question 2 (Q2)—What factors influence diabetic patients’ continuity of telehealth use over time?

Based on the theoretical foundation of UTAUT2, the following hypotheses were developed:

Hypothesis 1: eHealth-related characteristics would be positively associated with participation.

Hypothesis 2: socio-demographic factors would influence both participation and continuity.

Hypothesis 3: care frequency would be positively associated with continuity.

Hypothesis 4: eHealth literacy would moderate the relationship between care frequency and continuity.

### Measurement

#### Dependent variables.

In alignment with our research objectives, two key outcome variables were observed: participation willingness and continuity of telehealth services, both treated as binary categorical variables.

- For Question 1, participation willingness referred to the decision to join the telehealth program, measured at baseline (T1). Patients who agreed to participate were coded as 1, while those who declined were coded as 0 (analysed in Model 1).
- For Question 2, continuity reflected sustained engagement, defined by follow-up status at the program’s conclusion (T3) among those who joined the telehealth program. Participants who completed the program were coded as 1, and those who withdrew were coded as 0 (analysed in Model 2).

#### Independent variables and covariates.

A broad set of independent variables and covariates were assessed for their potential association with participation willingness and continuity. These included:

- **Socio-demographic characteristics**: gender, age, marital status, occupation, living status, self-rated economic condition, income source, and education level. Income source was self-reported and may therefore be subject to recall or social desirability bias.
- **Clinical characteristics**: diabetes type and diabetes duration.
- **Care frequency**: measured the extent of assistance accessibility within the household and was used as a pragmatic proxy for household assistance. This variable reflects the frequency of assistance rather than the quality, intensity, or type of caregiving and does not represent a validated caregiver support or caregiver burden scale.
- **Activity status**: assessed overall health functioning beyond diabetes.

Socio-demographic, clinical, care frequency, and eHealth-related variables were collected at baseline (T1), prior to participants’ decision regarding telehealth program enrollment. All baseline data were collected through structured questionnaires administered by trained research assistants during routine GOPC follow-up visits.

Socio-demographic characteristics included gender (self-reported as male or female), age (in years), marital status (single or married), occupation status (having or not having a full-time job), living status (living alone or with cohabitants), self-rated economic condition, income source, and highest education level attained. Clinical characteristics included diabetes type and duration of diagnosis. These variables were self-reported and recorded before telehealth participation was initiated.

### eHealth-related characteristics

- **Mobile use**: Number of years of mobile phone use was recorded.
- **Mobile technology confidence**: Measured using a single self-reported item assessing participants’ confidence in using a smartphone or mobile device. Responses were recorded on a 5-point Likert scale, ranging from “very little confidence” to “very confident,” with higher scores indicating greater perceived confidence in mobile technology use.
- **Health-related internet use**: Based on the US Health Information National Trends Survey (HINTS), participants reported engagement in five distinct internet activities for health-related purposes over the past 12 months. Each activity was coded as a binary variable (0 = no, 1 = yes), and a cumulative score ranging from 0 to 5 was computed, with higher scores indicating more diverse online health-related engagement [26,27].
- **eHealth literacy**: Assessed using the validated Chinese version of the eHEALS scale, which demonstrated excellent internal consistency (Cronbach’s alpha = 0.92) [28]. The scale consists of eight items rated on a 5-point Likert scale from “strongly disagree” to “strongly agree.” Total scores range from 8 to 40, with higher scores reflecting greater perceived eHealth literacy [28,29]. Specific operational definitions and coding details for all variables are summarized in Table 3.

### Variable selection and theoretical rationale

The selection of independent variables was guided by UTAUT2. This framework posits those broader constructs, such as Performance Expectancy, Effort Expectancy, Facilitating Conditions, and Social Influence, influence both behavioral intention and continued technology use. Accordingly, variables such as mobile technology confidence, eHealth literacy, health-related internet use, and care frequency were selected as indicators conceptually aligned with these theoretical constructs. Additional socio-demographic and clinical characteristics were also included, drawing on existing empirical literature related to telehealth uptake and chronic disease self-management.

To further explore the theoretical underpinnings of the Facilitating Conditions construct, we conceptualized an interaction term between eHealth literacy and care frequency. The rationale was to examine whether individuals with higher levels of eHealth literacy could better overcome limited household support and thus show higher continuity in telehealth engagement. This interaction reflects the hypothesis that eHealth literacy may act as a buffer or enhancer in the relationship between social-environmental factors and sustained use of telehealth technologies.

The interaction between eHealth literacy and care frequency was selected a priori based on theoretical relevance to the UTAUT2 construct of Facilitating Conditions and the hypothesized moderating role of digital capability in reducing reliance on household support. Other potential interaction terms were not examined to limit model complexity and avoid overfitting, particularly given the sample size of the telehealth subgroup. Accordingly, interaction analyses were restricted to theoretically driven hypotheses rather than exploratory testing of multiple interaction effects.

### Statistical analysis

#### Descriptive analysis.

Descriptive statistics were used to summarize the socio-demographic, clinical, and eHealth-related characteristics of the study population. Continuous variables were presented as means and standard deviations (SD), while categorical variables were summarized as frequencies and percentages. Group differences between participants and non-participants or those who continued versus discontinued telehealth were assessed using Pearson’s chi-square tests for categorical variables and independent t-tests for continuous variables. Descriptive comparisons were conducted for exploratory purposes and were not subjected to false discovery rate (FDR) correction; primary inferential conclusions were derived from the multivariable logistic regression models.

#### Modelling approach.

To address the two research questions, two binary logistic regression models were applied separately. Model 1 was conducted using the total sample (n = 1,094), while Model 2 was conducted using the telehealth group (n = 410). Results were reported as coefficients, along with odds ratios (ORs) and the corresponding 95% confidence intervals (CIs). For clarity, Model 1 and Model 2 are used throughout this manuscript to denote two analytic frameworks corresponding to the study research questions, rather than referring exclusively to individual regression equations. Model 1 encompasses analyses examining factors associated with participation willingness (Question 1), and Model 2 encompasses analyses examining factors associated with continuity of telehealth use (Question 2). Depending on context, these analyses include descriptive comparisons as well as multivariable logistic regression models. Analyses examining interaction effects within the telehealth subgroup were exploratory in nature, and findings were interpreted cautiously given the reduced sample size. Binary logistic regression was selected due to the categorical nature of the outcome variables and the study’s focus on identifying associated factors rather than modelling time-to-event or hierarchical effects.

#### Missing data and Model diagnostics.

Non-responders (n = 204)—those who declined to participate in the telehealth program after baseline data collection—were retained in Model 1 to assess factors associated with participation willingness. Participants with incomplete outcome data (<3%) were handled using multiple imputation to minimize potential bias. The proportion of missing outcome data was low (<3%). Multiple imputation was applied to minimize potential bias; given the minimal extent of missingness, no sensitivity analyses were conducted. The final response rate for telehealth program participation was 81.3% (890 out of 1,094 eligible patients). Multicollinearity among independent variables was assessed using the variance inflation factor (VIF), with values below 10 considered acceptable [30]. The Hosmer-Lemeshow goodness-of-fit test was conducted to evaluate model adequacy. All statistical analyses were conducted using IBM SPSS Statistics (Version 29). Statistical tests were two-tailed, and statistical significance was defined as p < 0.05.

## Results

Table 1 summarizes the socio-demographic, clinical, and eHealth-related characteristics of the study participants. A total of 1,094 participants were included in the analysis, with a mean age of 60.45 years (SD = 10.69). The majority were male (56.1%) and married (72.9%). Enrolled participants (mean age = 59.50) were, on average, younger than those who declined telehealth participation (mean age = 64.60). Additionally, a significantly higher proportion of men enrolled in the program (60.0% of enrollees vs. 39.2% of non-enrollees). Over 70% had completed middle school or higher education. More than half (59.0%) were not engaged in full-time employment. Regarding income sources, 62.9% relied on self-sustaining income, such as salaries and savings, while only 6.3% depended on pensions. Clinically, nearly all participants (97.8%) were diagnosed with Type 2 diabetes. The duration of diabetes varied: 38.3% had been diagnosed for 0–4 years, 21.0% for 5–8 years, and 40.7% for over nine years. In terms of eHealth-related characteristics, over 90% of participants had used smartphones for more than nine years. The average mobile technology confidence score was 3.83 (SD = 1.09), indicating moderate-to-high confidence levels. However, the mean score for health-related internet use was only 1.27 (SD = 1.21), reflecting low adoption for health purposes. The mean eHEAL score, which measures eHealth literacy, was 26.37 (SD = 7.85), indicating moderate levels of eHealth literacy (Table 2).

**Table 1:** Measurement of variables.

Name of variable	Variable type	Measurement
Gender	Binary nominal variable	0 = Male
		1 = Female
Age, (years)	Continuous variable	Years
Marital status	Binary nominal variable	0 = Single
		1 = Married
Occupation	Binary nominal variable	0 = No full time job
		1 = Have full time job
Living status	Binary nominal variable	0 = Living alone
		1 = Have cohabitant
Economics	Ordinal variable	1 = Very insufficient
		2 = Insufficient to meet daily expenses
		3 = Adequate to meet daily expenses
		4 = More than adequate
Income source	Nominal variable	1 = Self-sustaining income
		2 = Family support
		3 = Pension
		4 = Government assistance
Education	Ordinal variable	1 = No formal education
		2 = Primary school
		3 = Middle school and high school
		4 = Tertiary education and above
^a^DM type	Binary nominal variable	0 = Type 1
		1 = Type 2
^a^DM duration	Ordinal variable	1 = 0–4 years
		2 = 5–8 years
		3 = over 9 years
Activity status	Binary nominal variable	0 = Have restriction
		1 = No restriction
Care frequency	Continuous variable	Likert 5-point scale, with 5 indicating the highest frequency
Mobile using year	Ordinal variable	1 = 0–4 years
		2 = 5–8 years
		3 = over 9 years
Mobile technology confidence	Continuous variable	Likert 5-point scale, with 5 indicating the highest confidence
Health-related internet use	Continuous variable	Score from 0 to 5, with 5 representing the most frequently used
eHealth literacy	Continuous variable	Measured by the ^b^CeHEAL scale, with scores ranging from 8 to 40, where 40 represents the highest eHealth literacy

^^a^^ DM: Diabetes Mellitus

^b^ CeHEAL: Chinese version of eHealth Literacy Scale

**Table 2:** Descriptive statistic results.

Characteristic	Model 1				Model 2			
	Enroll (n = 890)	Unenroll (n = 204)	Total (n = 1094)	χ2 Statistics	Complete (n = 148)	Withdraw (n = 262)	Telehealth Group (n = 410)	χ2 Statistics
	**Count/%**				**Count/%**			
Gender				p < 0.001				p = 0.474
Male	534 (60.0%)	80 (39.2%)	614 (56.1%)		94 (63.5%)	157 (59.9%)	262 (63.9%)	
Female	356 (40.0%)	124 (60.8%)	480 (43.9%)		54 (36.5%)	105 (40.1%)	148 (36.1%)	
Age, (years)				p = 0.07				p < 0.001
Mean (SD)	59.50 (10.66)	64.60 (9.82)	60.45 (10.69)		55.57 (10.59)	59.40 (9.52)	58.02 (10.08)	
Marital status				p = 0.248				p = 0.658
Single	235 (26.4%)	62 (30.4%)	297 (27.1%)		41 (27.7%)	78 (29.8%)	119 (29.0%)	
Married	655 (73.6%)	142 (69.6%)	797 (72.9%)		107 (72.3%)	184 (70.2%)	291 (71.0%)	
Occupation				p < 0.001				p = 0.008
Have full-time job	402 (45.2%)	47 (23.0%)	449 (41.0%)		89 (60.1%)	122 (46.6%)	211 (51.5%)	
No full-time job	488 (54.8%)	157 (77.0%)	645 (59.0%)		59 (39.9%)	140 (53.4%)	199 (48.5%)	
Living status				p = 0.66				p = 0.968
Live alone	112 (12.6%)	28 (13.7%)	140 (12.8%)		19 (12.8%)	34 (13.0%)	53 (12.9%)	
Have cohabitants	778 (87.4%)	176 (86.3%)	954 (87.2%)		129 (87.2%)	228 (87.0%)	357 (87.1%)	
Economics				p = 0.298				p = 0.811
Very insufficient	8 (0.9%)	4 (2.0%)	12 (1.1%)		2 (1.4%)	2 (0.8%)	4 (1.0%)	
Insufficient to meet daily expenses	37 (4.2%)	13 (6.4%)	50 (4.6%)		8 (5.4%)	10 (3.8%)	18 (4.4%)	
Adequate to meet daily expenses	486 (54.6%)	108 (52.9%)	594 (54.3%)		83 (56.1%)	148 (56.5%)	231 (56.3%)	
More than adequate	359 (40.3%)	79 (38.7%)	438 (40.0%)		55 (37.2%)	102 (38.9%)	157 (38.3%)	
Income source				p < 0.001				p = 0.003
Self-sustaining income	601 (67.5%)	87 (42.6%)	688 (62.9%)		119 (80.4%)	172 (65.6%)	291 (71.0%)	
Family support	160 (18.0%)	75 (36.8%)	235 (21.5%)		16 (10.8%)	54 (20.6%)	70 (17.1%)	
Pension	58 (6.5%)	11 (5.4%)	69 (6.3%)		4 (2.7%)	23 (8.8%)	27 (6.6%)	
Government assistance	71 (8.0%)	31 (15.2%)	102 (9.3%)		9 (6.1%)	13 (5.0%)	22 (5.4%)	
Education				p < 0.001				p = 0.008
No formal education	18 (2.0%)	11 (5.4%)	29 (2.7%)		4 (2.7%)	2 (0.8%)	6 (1.5%)	
Primary school	183 (22.6%)	84 (41.2%)	267 (24.4%)		13 (8.8%)	45 (17.2%)	58 (14.1%)	
Middle school and high school	528 (81.9%)	91 (44.6%)	619 (56.6%)		88 (59.5%)	166 (63.4%)	254 (62.0%)	
Tertiary education and above	161 (18.1%)	18 (8.8%)	179 (16.4%)		43 (29.1%)	49 (18.7%)	92 (22.4%)	
DM^a^ type				p = 0.181				p = 0.887
Type 1	17 (1.9%)	7 (3.4%)	24 (2.2%)		2 (1.4%)	4 (1.5%)	6 (1.5%)	
Type 2	873 (98.1%)	197 (96.6%)	1070 (97.8%)		146 (98.6%)	258 (98.5%)	404 (98.5%)	
DM^a^ duration				p = 0.517				p = 0.598
0-4 years	344 (38.7%)	75 (36.8%)	419 (38.3%)		60 (40.5%)	97 (37.0%)	157 (38.3%)	
5-8 years	191 (21.5%)	39 (19.1%)	230 (21.0%)		30 (20.3%)	64 (24.4%)	94 (22.9%)	
over 9 years	355 (39.9%)	90 (44.1%)	445 (40.7%)		58 (39.2%)	101 (38.5%)	159 (38.8%)	
Activity status				p < 0.001				p = 0.210
Have restriction	38 (4.3%)	28 (13.7%)	66 (6.0%)		2 (1.3%)	9 (3.5%)	11 (2.7%)	
No restriction	852 (95.7%)	176 (86.3%)	1028 (94%)		148 (98.7%)	256 (96.5%)	404 (97.3%)	
Care frequency								p = 0.083
Mean (SD)			NA		4.37 (1.10)	4.17 (1.17)	4.24 (1.14)	
Mobile using year				p < 0.001				p = 0.329
0-4 years	29 (3.3%)	17 (8.3%)	46 (4.2%)		3 (2.0%)	2 (0.8%)	5 (1.2%)	
5-8 years	41 (4.6%)	19 (9.3%)	60 (5.5%)		1 (0.7%)	5 (1.9%)	6 (1.5%)	
over 9 years	820 (92.1%)	168 (82.4%)	988 (90.3%)		144 (97.3%)	255 (97.3%)	399 (97.3%)	
Mobile technology confident				p < 0.001				p = 0.074
Mean (SD)	3.99 (0.95)	3.16 (1.35)	3.83 (1.09)		4.17 (0.84)	4.02 (0.80)	4.07 (0.82)	
Health-related internet use				p < 0.001				p = 0.993
Mean (SD)	1.37 (1.22)	0.83 (1.06)	1.272 (1.21)		1.34 (1.23)	1.34 (1.24)	1.34 (1.24)	
CeHEAL^b^ score				p < 0.001				p = 0.018
Mean (SD)	27.48 (6.99)	21.48 (9.39)	26.37 (7.85)		29.59 (4.53)	28.36 (5.32)	28.80 (5.08)	
Participation (Model 1 DV)								
Willing			890 (81.4%)				NA	
Unwilling			204 (18.6%)				NA	
Completion (Model 2 DV)								
Complete			NA				148 (36.1%)	
Withdraw			NA				262 (63.9%)	

^a^ DM: Diabetes Mellitus

^b^ CeHEAL: Chinese version of eHealth Literacy Scale

Among the telehealth group, program completers (mean age = 55.57) were younger than those who withdrew (mean age = 59.40), and were slightly more likely to be male (63.5% vs. 59.9%).

Participants who received telehealth services (n = 410) exhibited characteristics similar to the overall sample but differed in certain areas. Participants who received telehealth services had a slightly younger mean age of 58.02 years (SD = 10.08). A higher proportion reported self-sustaining income (71.0%) compared to the overall cohort. Additionally, more than half (51.5%) were engaged in full-time employment. A larger proportion (84.4%) had completed middle school or higher education, reflecting higher educational attainment. Performance in eHealth-related measures, such as mobile technology confidence and eHEAL scores, was generally higher in the telehealth group.

Table 3 presents the results of the binary logistic regression analysis for both research questions. Model 1 explores the factors influencing participation willingness, while Model 2 examines the factors influencing continuity of telehealth services. Several estimates, particularly for economic subgroups, exhibited wide confidence intervals, likely reflecting small cell sizes; these results should therefore be interpreted with caution.

**Table 3:** Binary logistic regression models analysis results.

	Model 1		Model 2	
	Coefficient	Odds ratio (95% CI)	Coefficient	Odds ratio (95% CI)
**Gender**				
Female (ref. male)	-0.617	0.540 (0.369-0.789) ***	0.168	1.182 (0.698-2.003)
**Age**	-0.009	0.991 (0.969-1.014)	-0.031	0.969 (0.942-0.997) *
**Marital status**				
Married (ref. single)	0.139	1.149 (0.746-1.769)	0.397	1.487 (0.795-2.780)
**Occupation**				
No full time job (ref. have full time job)	-0.255	0.775 (0.475-1.263)	0.013	1.013 (0.566-1.815)
**Living status**				
Have cohabitants (ref. live alone)	0.015	1.015 (0.559-1.841)	-0.118	0.888 (0.391-2.020)
**Economics**				
Insufficient to meet daily expenses (ref. very insufficient)	0.916	2.498 (0.507-12.296)	0.763	2.145 (0.212-21.746)
Adequate to meet daily expenses (ref. very insufficient)	1.161	3.193 (0.760-13.426)	-0.003	0.997 (0.125-7.940)
More than adequate (ref. very insufficient)	0.925	2.521 (0.592-10.737)	-0.381	0.683 (0.085-5.468)
**Income source**				
Family support (ref. self-sustaining income)	-0.395	0.674 (0.419-1.084)	-0.919	0.399 (0.177-0.901) *
Pension (ref. self-sustaining income)	-0.049	0.953 (0.440-2.062)	-1.192	0.304 (0.090-1.022) *
Government assistance (ref. self-sustaining income)	-0.061	0.941 (0.503-1.763)	0.676	1.965 (0.658-5.868)
**Education**				
Primary school (ref. no formal education)	-0.425	0.654 (0.261-1.641)	-1.475	0.229 (0.033-1.605)
Middle school and high school (ref. no formal education)	-0.170	0.844 (0.330-2.159)	-1.296	0.274 (0.042-1.764)
Tertiary education and above (ref. no formal education)	-0.161	0.851 (0.291-2.486)	-0.858	0.424 (0.063-2.862)
**DM**^**a**^ **type**				
Type 2 (ref. Type 1)	0.666	1.946 (0.696-5.444)	0.090	1.094 (0.170-7.041)
**DM**^**a**^ **duration**				
5-8 years (ref. 0–4 years)	0.017	1.017 (0.633-1.635)	-0.252	0.778 (0.433-1.398)
over 9 years (ref. 0–4 years)	0.129	1.137 (0.769-1.683)	0.149	1.161 (0.683-1.974)
**Activity status**				
No restriction (ref. have restriction)	0.419	1.520 (0.807-2.865)	0.869	2.384 (0.416-13.653)
**Care frequency**	NA	NA	0.254	1.289 (1.024-1.621) *
**Mobile using year**				
5-8 years (ref. 0–4 years)	-0.422	0.656 (0.253-1.700)	-1.385	0.250 (0.012-5.431)
over 9 years (ref. 0–4 years)	-0.282	0.754 (0.361-1.576)	-1.464	0.231 (0.029-1.816)
**Mobile technology confident**	0.379	1.461 (1.238-1.722) ***	0.111	1.118 (0.832-1.502)
**Health-related internet use**	0.171	1.187 (1.003-1.404) *	-0.150	0.861 (0.707-1.047)
**CeHEAL**^**b**^ **score**	0.046	1.048 (1.022-1.074) ***	0.040	1.040 (0.988-1.095)
**Interactions between care frequency and CeHEAL**^**b**^ **score**			-0.049	0.952 (0.912-0.993) *

^a^ DM: Diabetes Mellitus

^b^ CeHEAL: Chinese version of eHealth Literacy Scale

For Model 1, female participants were significantly less likely to participate in the telehealth program compared to males (β = -0.617, OR = 0.540, 95% CI: 0.369–0.789, p < 0.001). This indicates that women had 46% lower odds of enrolling in the program than men. eHealth-related characteristics significantly influenced participation. Each one-point increase in mobile technology confidence was associated with a 46% increase in the odds of participation (OR = 1.461, 95% CI: 1.238–1.722, p < 0.001), suggesting digital self-efficacy plays a key role in willingness to use telehealth. Greater diversity in health-related Internet use was positively associated with participation (β = 0.171, OR = 1.187, 95% CI: 1.003–1.404, p < 0.05), indicating that patients who engaged in more online health-related activities were about 19% more likely to participate. Similarly, higher eHealth literacy was associated with increased likelihood of participation (β = 0.046, OR = 1.048, 95% CI: 1.022–1.074, p < 0.001), meaning that for each one-point increase in eHEALS score, the odds of participation rose by approximately 5%. Other socio-demographic and diabetes-related clinical factors were not statistically significant predictors of participation willingness.

For Model 2, older participants were less likely to continue using telehealth services (β = -0.031, OR = 0.969, 95% CI: 0.942–0.997, p < 0.05), suggesting that each additional year of age was associated with a 3.1% decrease in the odds of telehealth continuity. Income source was also a significant factor: compared to participants with self-sustaining income, those relying on pensions had substantially lower odds of continuing (β = -1.192, OR = 0.304, 95% CI: 0.090–1.022, p < 0.05), indicating a 70% reduction in likelihood. Participants relying on family support also had reduced odds (β = -0.919, OR = 0.399, 95% CI: 0.177–0.901, p < 0.05), representing a 60% lower likelihood of continuity. Higher care frequency was associated with greater telehealth continuity (β = 0.254, OR = 1.289, 95% CI: 1.024–1.621, p < 0.05), with each additional unit of care frequency corresponding to a 29% increase in the odds of continuing. Other variables were not statistically significant.

**Fig 3:**
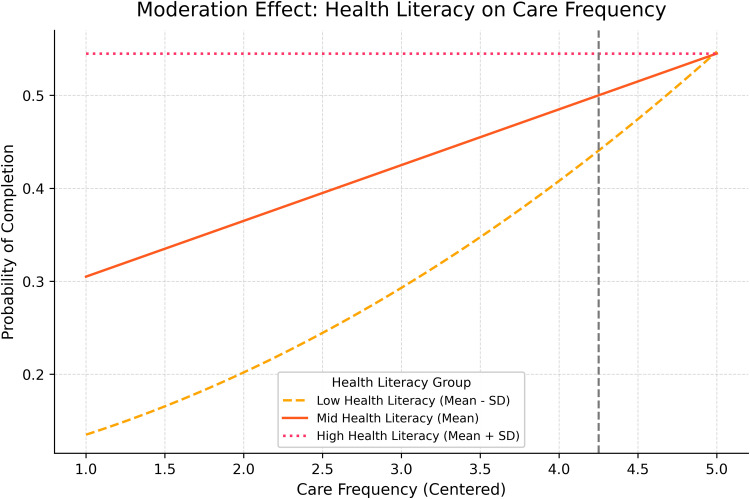
Illustration of the moderation effect of eHealth literacy on care frequerncy, care frequency was positively associated with telehealth continuity among participants with lower eHealth literacy. However, this positive association diminished as eHealth literacy increased.

A significant interaction was observed between care frequency and eHealth literacy (β = −0.049, OR = 0.952, 95% CI: 0.912–0.993, p < 0.05) as illustrated in Fig 3.

These results support Hypotheses 1–4, demonstrating the importance of eHealth-related characteristics, socio-demographics, care frequency, and their interaction in predicting participation and sustained telehealth use.

## Discussion

This study aimed to explore the factors influencing participation and continuity in telehealth services among diabetic patients in primary healthcare settings. The results demonstrate that socio-demographic characteristics such as gender, age, income source, care frequency, and eHealth related characteristics significantly influence both the initial participation and continuity of telehealth services.

Age emerged as a consistent barrier to both participation and continuity in telehealth. Prior studies indicate that older adults often experience challenges related to digital unfamiliarity, privacy concerns, and limited access to technology, particularly among those with reduced household support such as support from offspring or partners [14,15]. In the Hong Kong context, local research has similarly identified a persistent age-related digital divide, whereby older adults demonstrate lower digital skills and confidence in using information and communication technologies despite widespread digital infrastructure [31,32]. These locally documented disparities provide important contextual support for our findings and suggest that age-related barriers to telehealth use in Hong Kong are shaped by both individual digital capability and broader structural factors. These findings underscore the need for age-sensitive eHealth strategies tailored to elderly users.

Gender disparities were observed in telehealth participation, with female participants being less likely to enrol in the program. This disparity may reflect underlying differences in socio-demographic and digital characteristics between men and women rather than gender itself acting as a direct mechanism. Previous research has shown that women may experience disadvantages in employment status, lower digital confidence, and lower levels of eHealth literacy, all of which were observed to be associated with telehealth participation in our study [33]. These differences may partially account for the gender disparities observed in program enrolment. Evidence from Asian contexts suggests that gender differences in digital health adoption are context-dependent. A study from China [34] has reported gender differences in mobile health adoption, and regional analyses have highlighted persistent gender gaps in digital access and technology use across parts of Asia. These regional patterns provide contextual support for our findings and indicate that gender disparities in telehealth use may reflect structural differences in digital access and engagement rather than universal trends observed in Western settings [35]. Age was also associated with telehealth engagement, with older participants showing lower continuity over time. These findings indicate differential uptake across demographic groups; however, the underlying reasons for these patterns cannot be determined from the current data. This study did not collect qualitative information on patient preferences, perceived barriers, or decision-making processes related to telehealth participation. Accordingly, gender- and age-related differences should be interpreted as observed associations rather than explanatory mechanisms, and future mixed-methods research is needed to explore the contextual and experiential factors underlying these patterns.

Economic and occupational factors also shaped telehealth engagement. Participants with self-sustaining income (e.g., employment or personal savings) were more likely to adopt and continue using telehealth, potentially due to greater financial stability, autonomy in technology use, and flexible work schedules [36–38]. While economic status was not a significant predictor in multivariable models, descriptive trends suggested higher continuity among participants with full-time employment—likely because telehealth offers time-saving alternatives for working populations [39,40]. The self-rated nature of our economic measures may have underestimated these effects, highlighting the need for objective financial indicators in future studies.

Care frequency, used in this study as a proxy indicator of household support, was positively associated with telehealth continuity. This finding suggests that individuals who report more frequent household assistance may encounter fewer barriers to sustaining engagement, particularly for individuals who require support in managing digital tasks [41–43]. However, care frequency does not capture the nature, quality, or intentional involvement of caregivers, nor does it reflect caregiver decision-making or burden. As such, these findings should not be interpreted as evidence of caregiver integration into the telehealth intervention, but rather as an indication that the presence of household assistance may be relevant to continuity of use.

eHealth literacy emerged as a particularly strong determinant of both participation and continuity. Patients with higher eHealth literacy were more confident, capable, and autonomous in using telehealth platforms, consistent with prior studies [44,45]. Notably, eHealth literacy moderated the relationship between household support and continuity: among individuals with higher eHealth literacy, telehealth use was observed to be sustained with less household support, whereas those with lower literacy relied more heavily on family members’ involvement. This finding suggests that eHealth literacy may be associated with greater capability for independent engagement with telehealth services.

This interaction provides insight into potential mechanisms underlying telehealth continuity. Specifically, limited eHealth literacy may increase reliance on household support to maintain engagement, while higher literacy reduces such dependence [46]. This interpretation is consistent with intervention studies showing that technology education and digital literacy training can enhance telehealth uptake and sustained use. Together, these findings suggest that strengthening eHealth literacy and household support may play complementary roles in sustaining telehealth engagement, particularly among populations with lower digital capability.

Interpreted in relation to the UTAUT2 framework, the findings suggest that variables of mobile technology confidence and eHealth literacy, conceptually aligned with the Effort Expectancy and Facilitating Conditions were particularly associated with telehealth engagement in this primary care context. In contrast, variables such as income source, economics, occupation, and care frequency, conceptually related to Social Influence and Price Value, demonstrated weaker or more context-dependent associations. By context-dependent, we refer to the observation that the relative importance of UTAUT2-related constructs may vary according to population characteristics, care settings, and implementation environments. In routine primary care telehealth for chronic disease management, digital capability and perceived ease of use may outweigh social or economic considerations in shaping engagement. Importantly, this study does not test, validate, or modify the UTAUT2 model, but applies it conceptually to support theory-informed interpretation of observed associations. The results therefore highlight how certain UTAUT2 constructs may carry different relative importance in routine primary care telehealth settings, without implying structural extensions to the model.

Based on these associations, our findings suggest potential directions for intervention design, though these specific strategies were not directly tested in the current study. For instance, the strong link between eHealth literacy and participation suggests that future telehealth programs might benefit from age-sensitive onboarding (e.g., personalized coaching for older users), simplified app interfaces, and low-tech “starter kits” with instructional guides or video walkthroughs. Interventions such as digital literacy boot camps offered at clinics or community centres could be explored as methods to accelerate adoption, particularly in underserved populations. Additionally, providing optional household support or guidance for patients who rely on assistance for digital tasks may help address the barriers identified in our study. Future interventional research is required to empirically test the efficacy of these proposed strategies in improving telehealth uptake.

From a policy perspective, structural support is essential. Policymakers should consider subsidizing mobile data plans, internet access, or telehealth devices for low-income, elderly, or digitally excluded patients. These strategies align with global frameworks such as the WHO’s Digital Health Strategy and the Sustainable Development Goals (SDGs), which emphasize equitable access to eHealth solutions. Integrating eHealth literacy into chronic disease management policy can close critical access gaps and reduce long-standing health disparities.

Building on this, telehealth programs should embed digital literacy as a core component of chronic disease care, especially for populations with lower autonomy. Co-designing tools with patient, offering culturally adapted interfaces, and ensuring ongoing technical support can bridge the digital divide [47–50]. These efforts may support older adults and other individuals who rely on household assistance, while empowering patients to take an active role in their healthcare [51].

## Limitations

Several limitations should be acknowledged. First, the cross-sectional design is a critical constraint that limits causal inference; for example, we cannot determine whether mobile technology confidence leads to increased telehealth use, or if participation in the program builds that confidence. Longitudinal studies are needed to disentangle these temporal relationships. Second, the reliance on self-reported data for income, care frequency, and digital behaviour introduces potential bias. Specifically, variables such as income source and digital skills may be subject to recall bias or social desirability bias, potentially overestimating participants’ actual digital capabilities. Future research should incorporate objective digital usage metrics and verified financial indicators to validate these findings. Finally, the inclusion of both willing and unwilling participants created missing data on some follow-up variables. Addressing this with more complete longitudinal tracking and qualitative interviews could yield deeper insights into patient decision-making.

## Conclusion

In sum, this study reveals that, while telehealth offers promising alternatives for diabetes management, disparities in digital access, household support, and literacy persist. Tailored interventions—ranging from age-sensitive digital training to family members’ involvement—may bridge these gaps. Integrating eHealth literacy programs into primary care settings may empower patients with the skills and confidence to navigate telehealth tools, improving engagement and long-term management of chronic conditions. Future telehealth strategies must prioritize inclusivity, usability, and support structures to ensure successful transition to sustainable, person-centred digital care.

## Supporting Information

S1 STROBE Checklist STROBE Checklist. (DOC)
